# *Listeria monocytogenes* bacteremia developed during treatment of COVID‐19

**DOI:** 10.1002/ccr3.8115

**Published:** 2023-10-29

**Authors:** Jin Yamamoto, Keitaro Nakamoto, Teppei Shimasaki, Daisuke Kurai, Takeshi Saraya, Haruyuki Ishii

**Affiliations:** ^1^ Department of Nephrology and Rheumatology Kyorin University School of Medicine Mitaka Tokyo Japan; ^2^ Department of Respiratory Medicine Kyorin University School of Medicine Mitaka Tokyo Japan; ^3^ Department of General Medicine Kyorin University School of Medicine Mitaka Tokyo Japan

**Keywords:** bacteremia, COVID‐19, *Listeria monocytogenes*

## Abstract

*Listeria monocytogenes* is an important pathogen in older patients and immunosuppressed patients, often causing bacteremia. Complications resulting from infections other than COVID‐19 must also be considered during COVID‐19 treatment.

## Graphical abstract

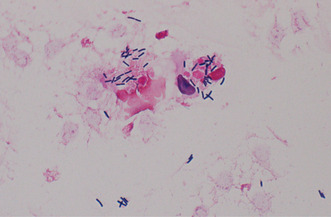

## 1 INTRODUCTION

*Listeria monocytogenes* is a Gram‐positive, nonspore‐forming, facultatively anaerobic coccobacillus.^1^ This important foodborne pathogen is one of the causative organisms in several outbreaks of foodborne disease. *L. monocytogenes* is an important pathogen in older patients, pregnant women, and immunosuppressed patients, often causing bacteremia and meningoencephalitis.^2^ The common symptoms of listeriosis include fever, headache, confusion, diarrhea, and abdominal pain. Coronavirus disease 2019 (COVID‐19) caused by severe acute respiratory syndrome coronavirus 2 (SARS‐CoV‐2) has been pandemic in Japan since 2020.^3^ Patients with risk factors such as advanced age are more likely to develop severe COVID‐19.^4^ However, multicenter study from the United Kingdom which enrolled total number of 48,902 COVID‐19 patients had no case of Listeriosis and we found only once case of co‐infected with Listeriosis from Pakistan.^5^, ^6^ We report a case of *L. monocytogenes* bacteremia that developed during treatment for COVID‐19.

## 2 CASE PRESENTATION

An 82‐year‐old man was seen at hospital complaining of persistent dyspnea for 4 days. His medical history included sarcoidosis, complete atrioventricular block, diabetes mellitus, prostate cancer, atrial fibrillation, and chronic kidney disease. He was taking prednisolone 5 mg/day for sarcoidosis and edoxaban 15 mg/day to prevent thrombosis and had received three vaccinations for COVID‐19. He drank a glass of wine a day and habitually consumed dry‐cured ham and cheese. On examination, his body temperature was 35.5°C, pulse rate 72/min, blood pressure 108/55 mmHg, and respiratory rate 24/min, with a saturation of peripheral oxygen (SpO_2_) of 92% on room air. His lungs were clear on auscultation.

**FIGURE 1:**
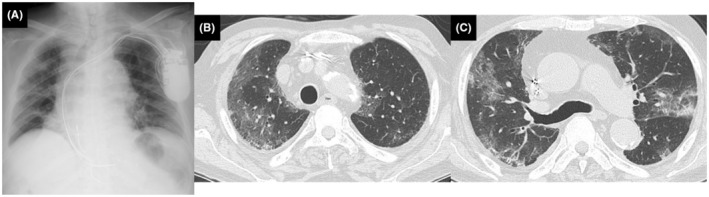
Chest x‐ray showed faint infiltrates in the bilateral lungs (A). Chest computed tomography showed bilateral ground glass opacities or consolidations predominantly in the subpleural region (B, C).

Laboratory findings showed an elevated neutrophil count (6.5 × 10^9^/L) and C‐reactive protein (CRP) level (235.7 mg/L). Chest x‐ray showed faint infiltrates in the bilateral lungs (Figure 1A). Chest computed tomography (CT) showed bilateral ground glass opacities or consolidations (Figure 1B,C). No significant bacteria were found in sputum or blood cultures. However, SARS‐CoV‐2 was detected from a nasopharyngeal swab by a BioFire® FilmArray Respiratory PCR Panel 2.1 plus (BioMérieux Japan Ltd., Tokyo, Japan).

**FIGURE 2:**
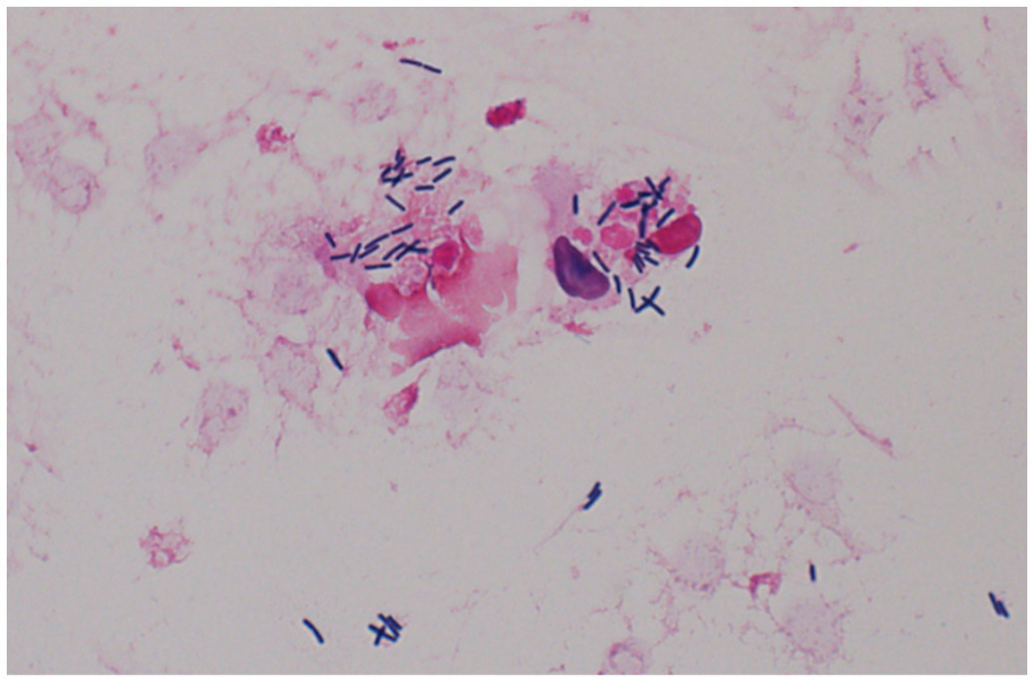
Gram stain in blood culture showed Gram‐positive short bacilli that were isolated and identified as *Listeria monocytogenes*.

**FIGURE 3:**
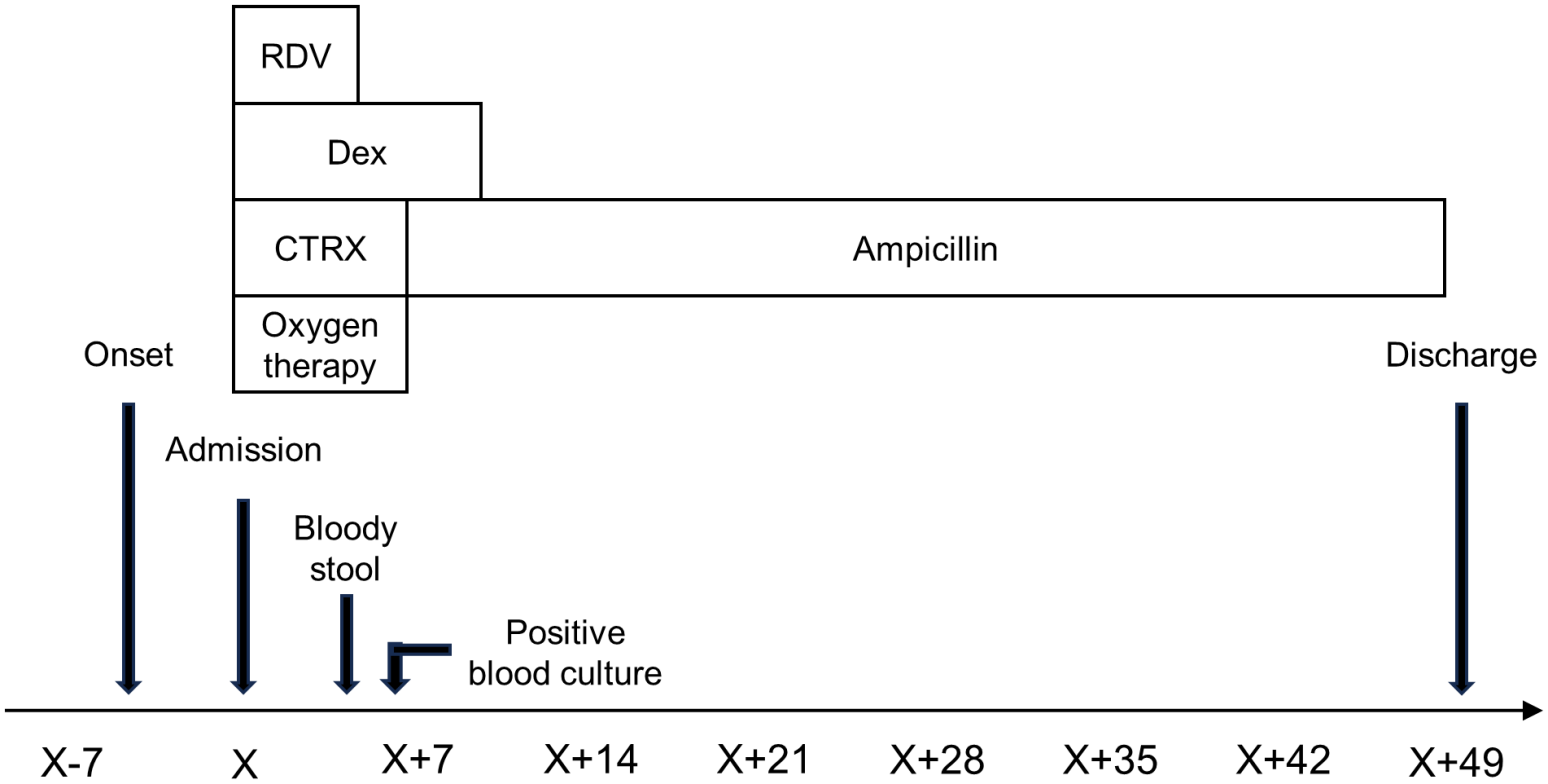
Clinical course in this case. CTRX, ceftriaxone; Dex, dexamethasone; RDV, remdesivir.

We diagnosed COVID‐19 pneumonia of moderate severity and hospitalized him. Infusion therapy with remdesivir (200 mg on Day 1, followed by 100 mg daily to 4 days) and dexamethasone (6 mg once daily for 10 days) and oxygen therapy were started, with ceftriaxone (2 g/day) administered for potential complications of bacterial pneumonia. Edoxaban, which he had originally been taking internally, was continued to prevent thrombosis. Four days after admission, bloody stools were observed, but the amount was small and not considered urgent. With treatment, his COVID‐19 symptoms and SpO_2_ level improved, and his body temperature remained at 36°C, but inflammation as indicated by CRP levels did not decrease. Seven days after admission, blood cultures were rerun, and Gram‐positive short bacilli were immediately cultured and considered to be *L. monocytogenes* based on results of matrix‐assisted laser desorption/ionization time‐of‐flight mass spectrometry and beta hemolysis on blood agar (Figure 2). We diagnosed *Listeria* bacteremia and changed to infusion therapy with ampicillin (8 g/day). Contrast‐enhanced head and trunk CT was performed, along with echocardiography, but no obvious abscess was found. After 42 days of ampicillin treatment, the inflammatory response had decreased, the patient was in good general condition, and he was discharged (Figure 3).

## 3 DISCUSSION

To our knowledge, this is the first case of *L. monocytogenes* bacteremia co‐occurring with COVID‐19 in Japan, similar to that reported from Pakistan.^6^ Russell et al. reported that bloodstream infections were most frequently caused by *Escherichia coli* and *Staphylococcus* aureus in immunocompromised COVID‐19 patients, but no case with listeria bacteremia was identified from their multicenter cohort study.^5^

*L. monocytogenes* is an important bacterium in older people and immunosuppressed patients as it can sometimes lead to serious conditions.^2^ Because large‐scale outbreaks are few, there are many unknowns regarding the route of infection and epidemiological background. However, an outbreak of cases caused by coleslaw in Canada in 1981 proved food to be one source of infection.^7^ No large‐scale outbreaks have occurred in Japan, foodborne listeriosis has been reported.^8^ The present patient had no common symptoms of *L. monocytogene*s‐related gastroenteritis such as fever, watery diarrhea, nausea, headache, and joint and muscle pains.^9^ Gastroenteritis typically occurs 24 h after ingestion (ranging from 6 to 240 h) of a large inoculum of *L. monocytogenes* and usually lasts 2 days. Although the occurrence of bloody diarrhea on the fourth hospital day would not rule out gastroenteritis associated with *L. monocytogenes*, it would be reasonable to also consider edoxaban. Moreover, common gastrointestinal symptoms of COVID‐19 are abdominal pain and diarrhea^4^ but rarely bloody stool.

Goulet et al. reported that the incubation period differed significantly based on the clinical form of invasive listeriosis.^10^ A longer incubation period was observed in pregnancy‐associated cases (median: 27.5 days; range: 17–67 days; interquartile range [IQR]: 20–36 days) than for meningo‐encephalitis cases (9 days; 1–14 days; IQR: 4–13 days) and bacteremia cases (2 days; 1–12 days; IQR: 1–5 days). Thus, bacteremia appearing on the seventh hospital day seemed to be caused by his habitual food intake. Although our patient’ s stool was not cultured, Hafner et al. reported that *L. monocytogenes* is detected in 10% of human stool samples from an independent cohort of 900 healthy asymptomatic donors, which might be applied to our patient due to his eating potentially contaminated products (ham and cheese).^11^ No cases caused by environmental contamination have occurred in our hospital, including in the high‐care unit/intensive care unit, for two decades, so this possibility is considered extremely low.

This case was consistent with several risks for listeriosis as the patient was older, had diabetes mellitus, was undergoing glucocorticoids therapy, and habitually ate cheese and other food items. In addition to the diversification of food, the distribution of cold foods has allowed long‐term preservation of foods, which may be one reason why cases of listeriosis have become common in Japan. Charlier et al. reported that prognostic factors associated with 3‐month mortality in patients with bacteremia and neurolisteriosis were ongoing cancer, aggravation of any preexisting organ dysfunction, multi‐organ failure, and monocytopenia.^2^ Our patient survived despite having two such risk factors (prostate cancer and renal dysfunction).

Listeriosis can affect other organs, with meningoencephalitis being a particularly serious complication.^12^ Because our patient showed no disturbance of consciousness and was taking an antithrombotic agent, we did not examine cerebrospinal fluid. Head CT examination was performed at a later date, but no obvious lesions were observed. Penicillin and ampicillin are effective treatments for listeriosis, and their concomitant use with gentamicin is also reported.^13^ Therefore, we changed from ceftriaxone to ampicillin based on the blood culture results. The patient was discharged from hospital after 6 weeks administration. He has refrained from taking cheese and certain other foods and has experienced no recurrence.

COVID‐19 has several risk factors for severe disease, which include older age, male sex, cardiovascular disease, chronic respiratory disease, diabetes, and obesity.^4^ Our patient also was older, a steroid user, and had diabetes mellitus. Currently, COVID‐19 treatment includes antiviral agents (remdesivir, nirmatrelvir/ritonavir, and molnupiravir) and neutralizing antibodies.^14^ As this patient had COVID‐19 pneumonia with respiratory failure, we used both remdesivir, which is approved in Japan, and dexamethasone, which is reported to reduced mortality in hospitalized patients with COVID‐19.^15^

The causes of complications of listeriosis are unclear, but the risk of bacterial infection is present because of the increased use of steroids in COVID‐19 treatment. Further, our patient was also at risk of bleeding from his oral antithrombotic medications. We thought that our patient's episode of bloody stools after admission had caused *L. monocytogenes*, which had been lurking in his intestinal tract, to enter the bloodstream. Furthermore, because COVID‐19 is highly infectious, our patient was treated in a private hospital room, and the increased stress of not being able to leave his room may have been an additional factor contributing to the gastrointestinal hemorrhage.

## 4 CONCLUSION

We report a case of *L. monocytogenes* bacteremia in a patient with COVID‐19. Careful history taking and recognition of immune status with proper timing of blood culture can lead to identify the co‐infected atypical organisms.

## AUTHOR CONTRIBUTIONS

**Jin Yamamoto:** Conceptualization; data curation; writing – original draft; writing – review and editing. **Keitaro Nakamoto:** Conceptualization; data curation; visualization; writing – original draft; writing – review and editing. **Teppei Shimasaki:** Supervision. **Daisuke Kurai:** Supervision. **Takeshi Saraya:** Conceptualization; supervision; writing – original draft; writing – review and editing. **Haruyuki Ishii:** Conceptualization; supervision.

## FUNDING INFORMATION

None.

## CONFLICT OF INTEREST STATEMENT

The authors declare no conflicts of interest associated with this article.

## CONSENT

Written informed consent was obtained from the patient for publication of this case report and accompanying images.

## Data Availability

The data that support the findings of this study are available on request from the corresponding author. The data are not publicly available due to privacy or ethical restrictions.
